# Differential Muc2 and Muc5ac secretion by stimulated guinea pig tracheal epithelial cells *in vitro*

**DOI:** 10.1186/1465-9921-7-35

**Published:** 2006-02-25

**Authors:** Brian N Chorley, Anne L Crews, Yuehua Li, Kenneth B Adler, Michael Minnicozzi, Linda D Martin

**Affiliations:** 1 North Carolina State University, College of Veterinary Medicine, Raleigh, NC, USA; 2 Schering Plough Research Institute, Kenilworth, NJ, USA

## Abstract

**Background:**

Mucus overproduction is a characteristic of inflammatory pulmonary diseases including asthma, chronic bronchitis, and cystic fibrosis. Expression of two mucin genes, *MUC2 *and *MUC5AC*, and their protein products (mucins), is modulated in certain disease states. Understanding the signaling mechanisms that regulate the production and secretion of these major mucus components may contribute significantly to development of effective therapies to modify their expression in inflamed airways.

**Methods:**

To study the differential expression of Muc2 and Muc5ac, a novel monoclonal antibody recognizing guinea pig Muc2 and a commercially-available antibody against human MUC5AC were optimized for recognition of specific guinea pig mucins by enzyme-linked immunosorbent assay (ELISA), Western blot, and immunohistochemistry (IHC). These antibodies were then used to analyze expression of Muc2 and another mucin subtype (likely Muc5ac) in guinea pig tracheal epithelial (GPTE) cells stimulated with a mixture of pro-inflammatory cytokines [tumor necrosis factor-α (TNF-α), interleukin 1β (IL-1β), and interferon- γ (IFN-γ)].

**Results:**

The anti-Muc2 (C4) and anti-MUC5AC (45M1) monoclonal antibodies specifically recognized proteins located in Muc2-dominant small intestinal and Muc5ac-dominant stomach mucosae, respectively, in both Western and ELISA experimental protocols. IHC protocols confirmed that C4 recognizes murine small intestine mucosal proteins while 45M1 does not react. C4 and 45M1 also stained specific epithelial cells in guinea pig lung sections. In the resting state, Muc2 was recognized as a highly expressed intracellular mucin in GPTE cells *in vitro*. Following cytokine exposure, secretion of Muc2, but not the mucin recognized by the 45M1 antibody (likely Muc5ac), was increased from the GPTE cells, with a concomitant increase in intracellular expression of both mucins.

**Conclusion:**

Given the tissue specificity in IHC and the differential hybridization to high molecular weight proteins by Western blot, we conclude that the antibodies used in this study can recognize specific mucin subtypes in guinea pig airway epithelium and in proteins from GPTE cells. In addition, Muc2 is highly expressed constitutively, modulated by inflammation, and secreted differentially (as compared to Muc5ac) in GPTE cells. This finding contrasts with expression patterns in the airway epithelium of a variety of mammalian species in which only Muc5ac predominates.

## Background

In the mammalian airway, mucus secreted by the epithelium and submucosal glands provides a defensive barrier between the outside environment and the airways. Mucus traps, neutralizes, and eliminates inhaled irritants, pollutants, and pathogens. Unfortunately, conditions that provoke overexpression of gel-forming mucin glycoproteins (the major structural components of mucus) can clog the conducting airways, and, ultimately, impair effective gas exchange. Many airway diseases, including asthma, chronic bronchitis, and cystic fibrosis, exhibit mucus overexpression [1-3]. Thus, understanding the mechanisms of expression and secretion of airway mucins has obvious pathophysiological significance and may assist in designing novel therapeutics for asthma and other airway diseases.

Airway mucins are derived from either epithelial goblet cells or epithelial cells of the submucosal gland [4]. At least twenty mucin genes have been reported, with expression of eight detectable in the human airway [5-9]. Four of these genes are known to encode gel-forming mucins (*MUC2*, *MUC5AC*, *MUC5B*, *MUC6*), while *MUC19 *was recently identified as having the potential to encode a gel-forming mucin based on its primary sequence [9]. MUC2 and MUC5AC expression are altered in inflamed airways [10-13] and, therefore, may contribute to the pathogenesis of several respiratory diseases. These mucins also exhibit cell- and tissue-specific expression in mammals where, in addition to their airway expression, Muc2 is expressed primarily in gastrointestinal epithelium and Muc5ac in gastric epithelium [14,15]. Differential regulation of mucin subtype expression may affect mucus composition in disease states, although little is known regarding mechanisms that modulate such expression [16-20].

The antigen-sensitized and -challenged guinea pig is an excellent model of allergic asthma, exhibiting major hallmarks of human asthma, including airway hyperresponsiveness and eosinophilic inflammation [21-24]. However, research using the guinea pig model has been hampered by the lack of available molecular tools, especially for studying mucin subtypes. Recently, Muc2 and Muc5ac-specific oligonucleotide probes were synthesized based on gene sequence information available from related mammalian species [25]. It was found that Muc2 gene expression increased with TNF-α stimulation in GPTE cells, whereas little, if any, Muc5ac mRNA expression was measured in control or stimulated cultures. Muc2 expression in airway epithelium is not commonly reported in other mammalian species, whereas Muc5ac is described frequently as the major gel-forming mucin in the airway epithelium of humans, horses and rodents [26-30].

The purpose of this study was to determine whether or not Muc2 and Muc5ac subtypes are regulated differentially in the guinea pig tracheal epithelium. A monoclonal antibody against Muc2 apomucin was developed for detection of guinea pig Muc2 and a commercially-available monoclonal antibody against human MUC5AC was optimized for detection of guinea pig Muc5ac. GPTE cells were exposed to a pro-inflammatory cytokine mix of TNF-α, IL-1β, and IFN-γ, and tested subsequently for differential expression of Muc2 and Muc5ac. While intracellular Muc2 and Muc5ac production and mRNA expression increased similarly, only apparent Muc2 secretion increased significantly over constitutive levels following inflammatory stimulation. These results demonstrate, for the first time, that mucin subtypes are regulated differentially in guinea pig tracheal epithelial cells, suggesting that different mechanisms may exist for mucin subtype storage and/or secretion.

## Methods

### Cell Culture

Primary cultures of differentiated GPTE cells were established using an air-liquid interface procedure [31]. Briefly, guinea pig tracheae were excised from euthanized animals, the epithelial cells dissociated proteolytically and washed. Transwell inserts (Corning Costar, Cambridge, MA) were coated with rat tail collagen type I (BD Biosciences, Franklin Lake, NJ) and the cells resuspended and seeded onto the inserts at a density of 5 × 10^4 ^cells/cm^2 ^in Dulbecco's modified Eagle's medium (DMEM)/F12 supplemented with 5% fetal bovine serum (FBS), 4 mM L-glutamine (Invitrogen, Carlsbad, California), 1% HL-1™ supplement, 25 ng/ml recombinant human epidermal growth factor (Serologicals, Norcross, GA), 50 nM retinal acetate, 100 μg/ml gentamicin, 40 U/ml nystatin (Sigma, St. Louis, MO), and 0.5 μg/ml amphotericin-B. Cells were cultured at 37°C in an atmosphere of 3% CO_2_. Medium was renewed every other day until the cells were 70–80% confluent (4–5 days), at which time medium was removed from the apical surface and cultures were fed basally with serum-free medium for an additional 7 days to allow mucous cell differentiation. Reagents listed above were purchased from Cambrex Corporation **(**East Rutherford, NJ), unless otherwise noted.

The Institutional Animal Care and Use Committee of North Carolina State University approved all protocols for the use of animals that pertain to this study.

### Production of anti-Muc2 monoclonal antibody

A 522 base pair fragment of guinea pig Muc2 cDNA, determined previously to be a coding region of the carboxy-terminal cysteine-rich region of guinea pig Muc2 [25], was cloned into a bacterial protein expression vector (pCAL-n; Stratagene, La Jolla, CA). The insert was verified by sequencing (DNA Sequencing Facility, University of North Carolina at Chapel Hill). The bacterial host strain BL21(DE3)pLysS (Stratagene, La Jolla, CA) was then transformed with the ligated vector. An overnight, ampicillin-selected BL21 (DE3)pLysS culture was expanded in Luria-Bertani broth at 37°C for 3–5 hrs with shaking. Protein expression was then induced by 1 mM isopropyl-1-thio-β-D-galactopyranoside (IPTG; Sigma) during logarithmic growth. Culture lysates of induced (+IPTG) and uninduced (-IPTG) cells were resolved on a 10% SDS-PAGE gel. Staining of total protein in the gel indicated high-level induction of a ~29 kDa protein, which formed an insoluble inclusion body. This protein fragment was purified by elution from an electrophoresis gel, using high concentrations of detergent to keep the inclusion body soluble. After elution, the putative Muc2 protein fragment was verified by SDS-PAGE and dialyzed into phosphate buffered saline (PBS), pH 7.4.

A monoclonal antibody was raised against this purified Muc2 protein fragment by the North Carolina State University Hybridoma facility. Specifically, a BALB/c mouse was inoculated 3 times with 100 μg of purified Muc2 protein fragment mixed with Freund's Incomplete Adjuvant over a 3 month time period. Serum from the mouse was collected two weeks after the final inoculation.

Antibody against the recombinant Muc2 protein fragment was detected in a 1:100 dilution of mouse serum collected from the antigen-challenged mouse via an ELISA (protocol below). Results indicated the presence of an anti-Muc2 antibody in the serum as a reaction was observed in test wells coated with 50 ng of purified Muc2 protein fragment. Three additional booster injections were administered to the mouse before antibody-producing spleen cells were collected and hybridized with murine myeloma cells (SP2/0-Ag14). Fused cells were diluted and plated to ~1 cell per well in eight 96-well plates and grown for two weeks in modified RPMI 1640 medium with 10% fetal bovine serum, 2 mM L-glutamine, 1× hypoxanthine-thymidine, 5 U/ml penicillin, and 5 μg/ml streptomycin at 37°C, 5% CO_2_. All culture reagents were purchased from Cambrex. Thirty-seven of 768 cultures were positive for Muc2 antibody when assayed by ELISA against purified Muc2 protein fragment. These cultures were expanded and retested. One clone (clone 4.68a) was selected for best overall performance based on immunohistochemistry, Western blot, and ELISA testing and was used for monoclonal selection and expansion.

Two additional rounds of small-scale expansion and selection were performed. Media containing secreted antibody from each clone was screened against the purified Muc2 protein fragment by ELISA. The hybridoma clone that secreted the antibody with the strongest immunoreactivity (clone C4) was selected for final large-scale expansion. Culture expansion was carried out in 225 cm^2 ^flasks (Corning Costar, Cambridge, MA) using the same media as above, with the exception that only 5% FBS was added in addition to 5% P388D1-derived growth supplement (American Type Culture Collection, Manassas, VA). The monoclonal antibody was purified using a Protein L column (Pierce, Rockford, IL) according to the manufacturer's instructions. Column fractions containing antibody were verified by ELISA, desalted and resuspended in 50% glycerol. The purified monoclonal antibody was isotyped as mouse IgM using a commercially available isotyping kit (Roche, Indianapolis, IN).

### Collection of guinea pig tissues and mucus

Multiple tissues were dissected from euthanized adult, male Hartley guinea pigs (Charles River, Stone Ridge, NY). For paraffin-embedded sections, tissue was placed in 10.0% formaldehyde, rested overnight, and then paraffin-embedded and sectioned.

Mucus secretions were also collected from small intestine, stomach and tracheal tissue samples. The internal epithelial surface of each tissue was exposed, rinsed repeatedly with PBS, and then scraped with a rubber policeman to remove mucus. Mucus was diluted (1:1) into PBS containing Complete Mini Protease Inhibitor Cocktail (Roche). Isolated secretions were stored at -80°C.

### Western blot of mucus proteins

Samples of isolated mucus secretions were solubilized in denaturing sample buffer [32]. This buffer contains a reducing agent reported previously to reduce thiols in the mucin thereby destroying epitopes that react with the 45M1 antibody [33]. We have tested human mucin samples with a wide range of reducing agent concentrations, and have found that the 45M1 antibody reacts with many of these samples in an ELISA format, suggesting the amount of reducing agent per amount of mucin is critical to obtaining a reaction with the antibody. Thus, we have included a reducing agent in our buffer, but have used more protein (23.5 μg) for our Western analyses then reported previously [32]. This enhances protein solubilization while keeping enough epitopes intact for antibody recognition.

Samples were then centrifuged at 10,000 rpm for 5 mins, and loaded onto a 1.0% agarose gel using a horizontal gel apparatus (Bio-Rad, Hercules, CA). Electrophoresis of samples was carried out at 15 V (1.9 V/cm gel) for 18 hrs. After the gel was equilibrated in tris/glycine buffer (Bio-Rad) for 30 mins, proteins were transferred to a nitrocellulose membrane using a semi-dry transfer apparatus (Bio-Rad) according to the manufacturer's instructions. The nitrocellulose membrane was washed in PBS and then blocked with 3% powdered milk in PBS. The membrane was hybridized with primary antibody (newly developed C4 or 45M1 from Lab Vision, Fremont, CA), at stated dilutions, in 1% milk at 4°C overnight. After washing the nitrocellulose membrane in PBS, the membrane was incubated with horseradish peroxidase (HRP)-conjugated goat anti-mouse antibody (MP Biomedicals, Irvine, CA) diluted 1:2000 in 1% milk. The membrane was then washed in PBS, washed additionally in PBS + 0.05% Tween-20, and finally rinsed with water. An ECL™ chemiluminescent detection kit (Amersham Biosciences, Piscataway, NJ) was used for visualization.

### Enzyme linked immunosorbent assay (ELISA)

A 96-well high-binding ELISA plate (Corning Costar) was coated with experimental samples at 4°C overnight. The plate was then washed twice with PBS. Wells were blocked with a solution of 3% cold-water fish gelatin (Sigma) in PBS for two hrs at room temperature. Following two washes with PBS, samples were exposed to the primary antibody (newly developed C4 or 45M1) diluted as indicated in 0.3% cold-water fish gelatin for 1 hr at room temperature. The plate was washed three times, labeled with a HRP-conjugated goat anti-mouse IgG (MP Biomedicals, Irvine, CA) diluted 1:2000 in 0.3% cold-water fish gelatin, and incubated for 1 hr at room temperature. After secondary antibody incubation, the plate was washed with PBS, and the color developed for 5 mins to 3 hrs, depending on primary antibody concentration and immunoreactivity. A 1M H_2_SO_4 _solution was then added to halt color development and absorbance was read at 450 nm.

### Immunohistochemistry

Paraffin-embedded sections of guinea pig liver and trachea were immunostained by the Histopathology Laboratory at the North Carolina State University, College of Veterinary Medicine. Sections of guinea pig lung tissue were provided by Dr. Allison Fryer of Oregon Health and Science University (Portland, OR) and were stained in our laboratory. Briefly, sections were deparaffinized in xylene, rehydrated through a series of graded ethanol solutions, rinsed with deionized water and endogenous peroxidase activity was blocked by a 10 min immersion in methanol containing 3% H_2_O_2_. After rinsing with ice-cold deionized water and then PBS, sections were blocked with normal goat serum for 20–30 mins., followed by application of primary antibody (C4 to detect Muc2 and 45M1 to detect Muc5ac) for 30 mins. A 1:50 dilution of C4, or a 1:100 dilution of 45M1, was used. Sections were rinsed with PBS and incubated with biotin-labeled goat anti-mouse antibody (Vector Labs, Burlingame, CA) for 20–30 mins. Sections were rinsed again with PBS, and incubated with streptavidin peroxidase complex for 10–20 mins. Sections were then rinsed with PBS, developed with 3, 3'diaminobenzidinetetrahydrochloride (DAB), and counterstained with hematoxylin. Sections were rinsed for 5 mins under tap water, dehydrated through a graded ethanol series and cleared with xylene prior to mounting. Negative controls were performed by substituting PBS for primary antibody.

### Real-time reverse transcriptase polymerase chain reaction (RT-PCR)

Total RNA was isolated from lysates of GPTE cells using the RNeasy Mini Kit (Qiagen, Valencia, CA) according to the manufacturer's instructions. Each sample was treated with RNase-free DNase for 15 mins to reduce DNA contamination. RNA with A_260_/A_280 _ratio of 1.95 or greater was used for reverse transcription. 1 μg of RNA from each sample was reverse transcribed in a 20 μl reaction using iScript cDNA synthesis kit (Bio-Rad) according to recommended conditions. 0.5 μl of each reverse-transcribed sample was added to 1× iQ SYBR Green Supermix (Bio-Rad), 200 nM of forward and reverse primers, and nuclease-free water to a 25 μl final reaction volume. Primer sequences and cycling conditions for Muc2, Muc5ac, and γ-actin were as described previously [25]. Amplifications were performed on an iCycler iQ Real-Time PCR Detection System (Bio-Rad). The starting amount of cDNA template was extrapolated from amplification curves by the method of Peirson [34]. Values reported are normalized to γ-actin levels and expressed as percentage of control. Melt-curve analysis was performed to verify that only a single product was amplified in each reaction.

### Exposure of guinea pig tracheal epithelial cells to cytomix

On day 7 after GPTE cell cultures were exposed to air, secreted mucus was removed from the apical surface with two washes (1× PBS). The basal media was changed and the cultures incubated at 37°C, 3% CO_2 _for 12 hrs at which time the secreted apical mucus was collected with a single PBS wash (0.5 ml). Mucins in these collections were measured by ELISA (see above) to determine BASELINE values for each culture. Basal media was again changed to begin the EXPERIMENTAL protocol. The EXPERIMENTAL protocol spanned an additional 12 hrs, during which time a pro-inflammatory cytokine mixture, "cytomix" (10 ng/ml each of TNF-α, IL-1β, and IFN-γ), was added to the culture media at 0, 4, and 8 hrs after EXPERIMENTAL time zero. In addition, control cultures were exposed to media only for the 12-hr EXPERIMENTAL period to allow measurement of CONSTITUTIVE mucus secretion. After the EXPERIMENTAL exposure period, mucus secretions were collected with a 0.5 ml PBS wash. BASELINE, EXPERIMENTAL and CONSTITUTIVE mucin samples for each culture well were assayed in duplicate on the same 96-well ELISA plate to minimize interplate variability. Undiluted mucus samples were always used in the ELISA unless otherwise noted. Normalized values were calculated by dividing the absorbance reading corresponding to mucin secreted for the EXPERIMENTAL period by the absorbance reading corresponding to mucin secreted during the BASELINE period, allowing each well to serve as its own control. Final EXPERIMENTAL/BASELINE results were expressed as a percentage of normalized CONSTITUTIVE mucin production for the 12-hr EXPERIMENTAL period. All mucin samples collected from GPTE cell cultures, unless otherwise noted, were treated with neuraminidase enzyme originating from *Arthrobacter ureafaciens *(Calbiochem, La Jolla, CA) by a method described previously [35].

### Statistical analysis

Experimental data were analyzed for significance using paired Student's *t *test or ANOVA with Tukey's post-test comparisons, where appropriate. Differences between treatments were considered significant at *p *< 0.05. Data are represented as mean ± standard error of the mean (SEM).

## Results

### C4 monoclonal antibody recognizes Muc2

**Figure 1:**
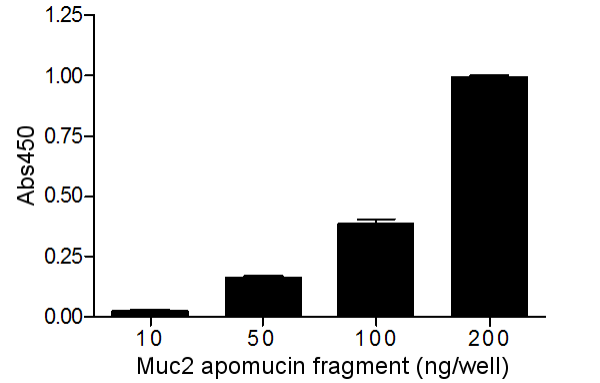
**Anti-Muc2 monoclonal antibody (C4) binds to Muc2 apomucin in ELISA. **Guinea pig Muc2 apomucin fragment (10, 50, 100, and 200 ng/well) purified from transformed bacterial culture lysate was measured by ELISA using purified C4 monoclonal antibody (400 μg/ml), diluted 1:100 in 0.3% cold-water fish gelatin. Results indicate the C4 monoclonal antibody binds to the Muc2 apomucin fragment in a concentration-dependent manner. Absorbance values read at 450 nm are shown corrected for background. Each sample was measured in triplicate, and data are shown as mean ± SEM. This assay was repeated three times with similar results.

To verify that the novel monoclonal antibody (C4) developed in this study would recognize guinea pig Muc2 apomucin, a dilution series of purified Muc2 protein fragment was examined by ELISA. C4 bound as little as 50 ng per test well, and absorbance values increased in a concentration-dependent manner (Fig. 1).

**Figure 2:**
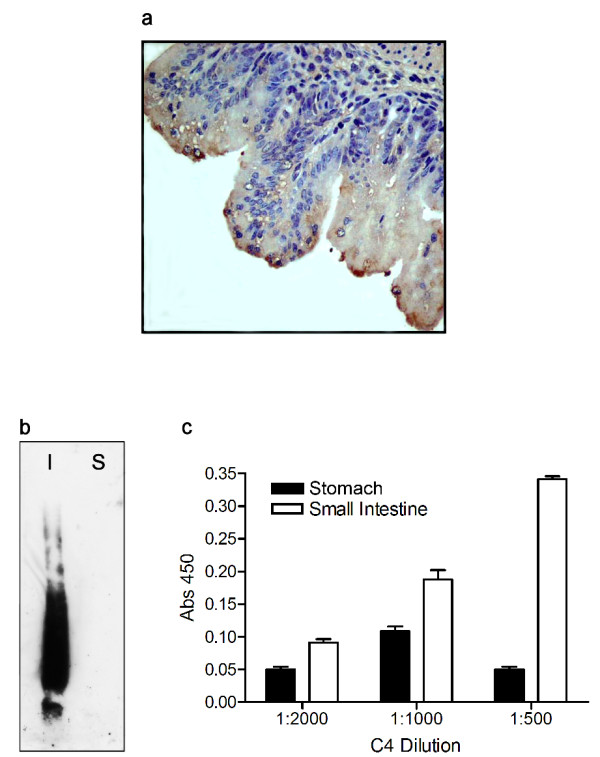
**C4 antibody recognizes small intestinal mucus preferentially by immunohistochemistry, Western blot, and ELISA. a) Immunohistochemistry. **The C4 antibody (diluted 1:50) recognizes the epithelial lining of fetal mouse small intestine by immunohistochemistry. Note dark brown staining of specific cells. Magnification using 40× objective. **b) Western blot hybridized with the C4 antibody. **23.4 μg of mucus collected from either the guinea pig small intestine (I) or stomach (S) was subjected to electrophoresis through a 1.0% agarose gel and then transferred to a nitrocellulose membrane. Hybridization with the C4 antibody (1:100) labeled a high molecular weight smear and a second high molecular weight band in the intestinal mucus, but did not recognize the stomach mucus. Western blot shown is representative of three separate blots. **c) ELISA **plate was coated with equal amounts of guinea pig stomach or small intestinal mucus (11.7 μg per well). The C4 antibody (1:500) preferentially recognizes the small intestinal mucus, although less differentiation is observed at lower working concentrations of the antibody. Each sample was assayed in triplicate, and data are shown as mean ± SEM. The assay was done twice using two independent sample isolations, with similar results obtained each time.

Given that C4 readily recognizes a Muc2 apomucin epitope, the ability of this antibody to recognize Muc2 was further tested by probing the mucin-rich mucosae of the small intestine using IHC. In mice, the small intestine is known to express a large amount of Muc2 while Muc5ac is not normally detected [15,36]. In mouse tissue sections, C4 stained the epithelial lining of the small intestine (Fig. 2a). Negative controls (C4 antibody on guinea pig liver; serum substitution for primary antibody on mouse intestine) showed no staining (data not shown). Thus, the C4 antibody appears to selectively recognize protein within tissues known to express large amounts of Muc2, such as the murine small intestine.

Western blot analysis was then used to verify specificity of the C4 antibody. C4 hybridized to mucus from the guinea pig small intestine but not from stomach (Fig. 2b). Specifically, the C4 antibody labeled two discernable bands: one fast mobility band and an intense, slower mobility band or smear. This dual banding pattern is similar to that described by Axelsson et al. [37] for mucus obtained from a colon adenocarcinoma cell line and immunoprecitiated with MUC2-specific antibodies. In that study, a faster mobility band was described as the MUC2 monomer, whereas a slower mobility band was thought to consist of MUC2 oligomers resistant to reduction treatment, a finding confirmed elsewhere [38].

To expand the utility of the C4 antibody for relative quantitation of specific mucins, we established conditions for its use in a more sensitive ELISA. C4 antibody bound mucus isolated from both guinea pig small intestine and stomach by ELISA (Fig. 2c), but at a 1:500 dilution of this antibody (0.8 μg/ml) small intestinal mucus was recognized selectively. Thus, we chose to use this dilution for all subsequent ELISAs, as it appeared to allow greater differential recognition of Muc2.

### 45M1 monoclonal antibody recognizes Muc5ac in guinea pig tissue

**Figure 3:**
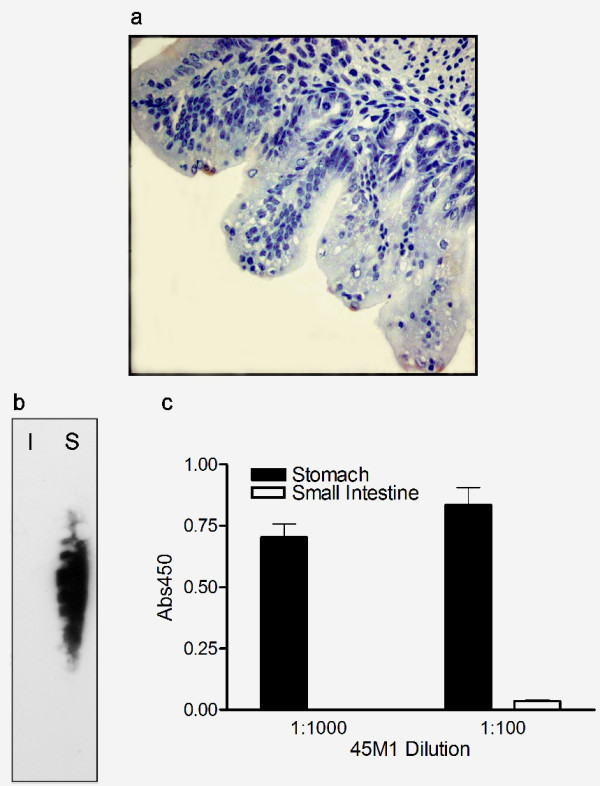
**45M1 antibody recognizes stomach mucus preferentially by Western blot, and ELISA. a) Immunohistochemistry. **The 45M1 antibody (diluted 1:100) does not recognize the majority of the cells lining a section of fetal mouse small intestine. Magnification using 40× objective. b) **Western blot hybridized with the C4 antibody. **23.4 μg of mucus collected from either guinea pig small intestine (I) or stomach (S) was subjected to electrophoresis through a 1.0% agarose gel and then transferred to a nitrocellulose membrane. Hybridization with the 45M1 antibody labeled high molecular weight protein in stomach mucus, with no labeling evident for small intestinal mucus. Western blot shown is representative of three separate blots. **c) ELISA **wells were coated with 5.8 μg of guinea pig stomach or small intestinal mucus. 45M1 (1:100 and 1:1000) recognized stomach mucus, but not small intestinal mucus. Each sample was assayed in triplicate and data are shown as mean ± SEM. The assay was done twice using two independent sample isolations, with similar results obtained each time.

To examine expression of Muc2 versus Muc5ac in guinea pig tissues, we developed guinea pig-specific assays using 45M1 (Lab Vision), a commercially-available antibody known to recognize Muc5ac in tissues from human, monkey, rat, rabbit, pig, and chicken [33,39]. Although Muc2 was detected in murine small intestinal tissue (Fig. 2a), a nearby section from this same tissue showed only minimal reaction to 45M1 (Fig. 3a). Negligible, non-specific labeling was observed when the 45M1 antibody was reacted to sections of guinea pig liver or when tissue sections were reacted with control mouse sera in place of the primary antibody (data not shown).

To further verify that the 45M1 monoclonal antibody could recognize Muc5ac from guinea pig with specificity, the antibody was hybridized to Western blots containing mucus from guinea pig stomach and small intestine (Fig. 3b). As anticipated, 45M1 recognized only the stomach sample. Specifically, a 1:5000 dilution of the 45M1 antibody labeled a smeared, high molecular weight band. This band was similar to those observed in Western blots containing respiratory secretions from normal or chronic bronchitic subjects [40] or samples of mucus secreted from cell lines of human mucosal origin [32]. Antibodies used in these earlier experiments were raised against peptide sequences corresponding to portions of MUC5AC; specifically, RNQDQQGPFKMC [40] or WFDVDFPSPGPHGGDKETYNNI [32].

Using 45M1 in an ELISA, a 1:1000 or 1:100 dilution of the antibody recognized 5.8 μg of total stomach mucus per well (Fig. 3c). The same dilutions of 45M1, however, did not bind to wells coated with an equal amount of total protein isolated from the small intestinal mucosa.

### C4 and 45M1 antibodies recognize specific cells in the guinea pig airway epithelium

**Figure 4:**
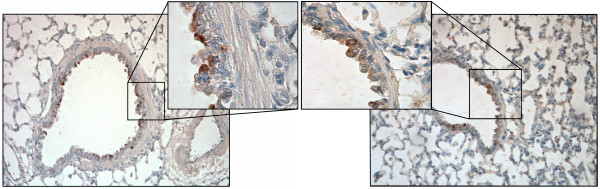
**Specific cells in the guinea pig airway epithelium are recognized by C4 and 45M1 antibodies. **IHC of guinea pig lung sections incubated with C4 (1:50; left panels) or 45M1 (1:100; right panels) antibodies. Insets are higher magnifications (400×) of the areas indicated by the squares in the reference micrographs (100× magnification). Note the distinctive staining of specific epithelial cells (dark brown) and the lack of staining in underlying cells and surrounding alveolar regions.

To determine whether the C4 and 45M1 antibodies could recognize proteins in the guinea pig lung *in vivo*, we subjected sections from guinea pig lung to immunohistochemistry employing these antibodies. Both antibodies recognized specific cells in the airway epithelium with little to no staining observed in underlying airway cells, nearby endothelium or in the alveolar regions (Fig. 4). Thus, these findings suggest the guinea pig airway epithelium expresses abundant Muc2 (Fig. 4, left panel) and Muc5ac (Fig. 4, right panel).

### Neuraminidase treatment improves 45M1 performance in ELISA

**Figure 5:**
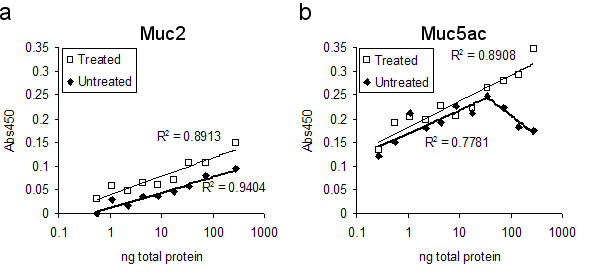
**Neuraminidase treatment enhances 45M1 performance in ELISA. **Mucin secretions were collected from GPTE cell cultures on day 9 after exposure to air. Samples were treated with neuraminidase (0.625 mU/μg total protein) for 2 hrs at 37°C [35]. Both neuraminidase-treated and untreated samples were diluted 2-fold in series, then coated on ELISA plates in duplicate. **a) **Neuraminidase treatment slightly increased absorbance values using C4 antibody (diluted 1:500), although the logarithmic slope and trend line fit of both treated and non-treated samples remained similar. **b) **Neuraminidase treatment improved the linear range of protein detection in an ELISA using the 45M1 monoclonal antibody (diluted 1:1000), as indicated by better R^2 ^value fit (calculated for ranges of increasing absorbance with increasing concentration) of the logarithmic trend line. Specifically, treatment extended the linear detection range above 35 ng/well.

Both the anti-Muc2 (C4) and the 45M1 antibodies are thought to preferentially recognize core mucin proteins. Since mucin populations isolated from lung are known to be heavily O-glycosylated [40,41], it is sometimes possible to enhance the specific performance of mucin-recognizing antibodies by removing carbohydrate moieties that obscure the protein backbones [35]. Neuraminidase, an enzyme that cleaves neuraminic acid carbohydrate groups from mucin glycoproteins [35], was used to determine whether recognition by the C4 or 45M1 antibodies could be enhanced in our ELISA method. Serially diluted samples of secreted mucus from GPTE cells, with and without neuraminidase treatment, were subjected to ELISAs using both mucin subtype monoclonal antibodies (Fig. 5). Neuraminidase treatment had minimal impact on C4 performance in ELISA (Fig. 5a), with both treated and non-treated curves having similar slope and R^2^-fit. Neuraminidase treatment had a greater impact on 45M1 ELISA performance (Fig. 5b), extending the linear range to higher concentrations. Without treatment, the 45M1 antibody recognized a linear relationship of protein concentration to absorbance for total mucus concentrations to 35 ng/well. With neuraminidase treatment, consistent increases in absorbance corresponding to increased mucus concentration were observed over the range of 0.07 to 280 ng/well. Based on these findings, all mucin collected from GPTE cells was subjected to neuraminidase treatment prior to ELISA to avoid potential underestimation of the amount of Muc5ac.

### Muc2 and Muc5ac are differentially secreted from GPTE cells

**Figure 6:**
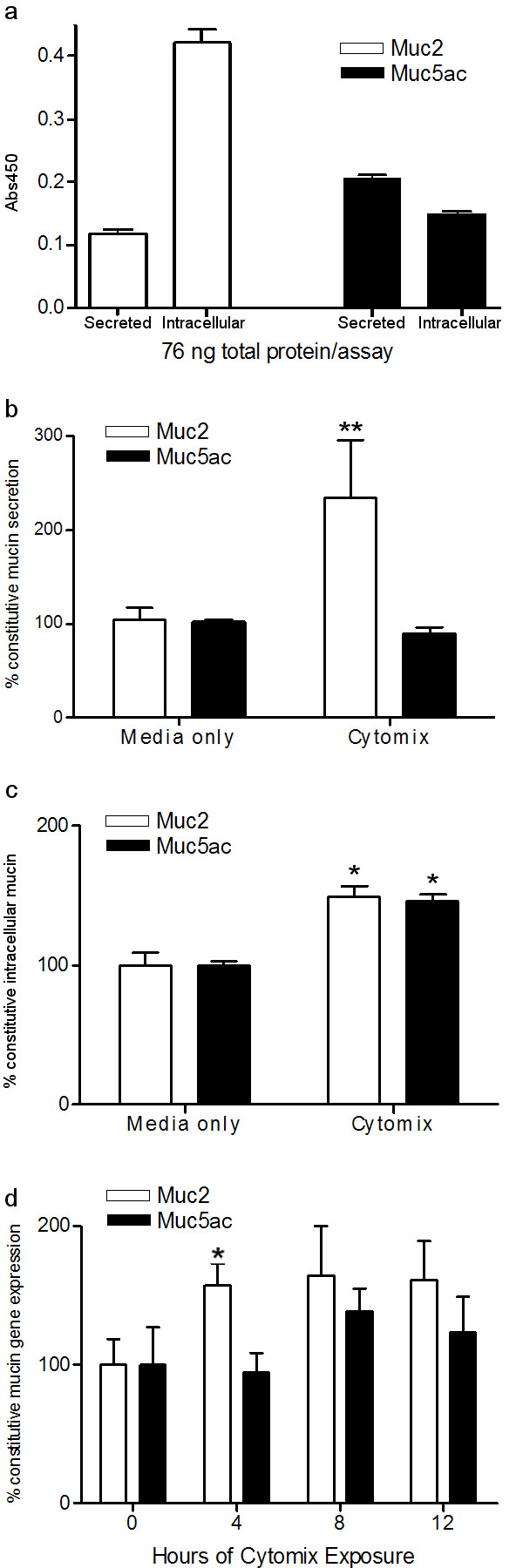
**Muc2, but not Muc5ac, secretion increases with pro-inflammatory stimulation in GPTE cells. a) **Total protein concentration of pooled samples of mucin collected from the apical surface or as intracellular lysates of non-stimulated GPTE cell cultures was determined using the Bradford assay. Equal amounts of protein were used to coat ELISA wells (76 ng/well), and then the presence of Muc2 or Muc5ac was examined. Results indicate that, in unstimulated GPTE cells, there is a higher ratio of intracellular to secreted Muc2 when compared to the ratio of intracellular to secreted Muc5ac. Samples from six cultures were pooled. Error bars represent assay variance of triplicate measurements. **b) **GPTE cells were exposed to air for 7 days during which time goblet cell differentiation occurred and mucus secretion began. Apical surfaces were washed, media changed, and then collected for secreted mucus baseline after 12 hrs incubation at 37°C, 5% CO_2_. Media was then changed and cultures were rested for 8 hrs and exposed to cytomix for 4 additional hrs. Relative amounts of Muc2 or Muc5ac in experimental and baseline collections were determined by ELISA. Experimental absorbance values were then corrected with baseline values, allowing each well to serve as its own control. These normalized values are expressed as a percentage of CONSTITUTIVE secretion observed during the 12-hr rest/exposure period, with the amount of Muc2 or Muc5ac secreted in cultures exposed only to media arbitrarily set at 100% of constitutive secretion. Results indicate Muc2 secretion is augmented by cytomix exposure, while Muc5ac secretion is equivalent in cytomix-treated and media-exposed (control) cultures. n = 5 in each group. ** = Significant change from constitutive mucin secretion by Student's *t*-test (p < 0.05). **c) **GPTE cells were exposed to cytomix or media (as a control) for 4 hrs. Then cells were lysed and assayed for intracellular mucin. The amount of Muc2 or Muc5ac in intracellular lysates from cells exposed only to media was arbitrarily set at 100%. In intracellular lysates from cells exposed to cytomix, both Muc2 and Muc5ac production was increased 50% over CONSTITUTIVE mucin levels. Subsequent time points (8 or 12 hrs exposure) showed a return to constitutive intracellular levels (data not shown). n = 6 in each group. * = Significant change from constitutive mucin production by Student's *t*-test (p < 0.01). **d) **GPTE cultures were exposed to 4, 8, and 12 hrs of cytomix. Total RNA was collected and Muc2 and Muc5ac transcript levels were determined using real-time RT-PCR. At 4 hrs, Muc2 mRNA levels were significantly increased when compared to constitutive expression. A trend toward an increase in both Muc2 and Muc5ac mRNA levels following cytomix exposure when compared to constitutive expression was observed at later time points. n = 4–6 in each group. * = Significant change from constitutive mucin production by Student's *t*-test (p < 0.05). All data points are presented as mean ± SEM.

Finally, we sought to determine whether specific mucin subtypes were expressed differentially in GPTE cells. To examine constitutive Muc2 and Muc5ac secretion versus intracellular expression, equal amounts of total protein derived from extracellular secretions or intracellular lysates were quantified for both mucin subtypes by ELISA (Fig. 6a). The relative amounts of Muc2 and Muc5ac glycoproteins cannot be determined directly by this approach due to lack of a coordinated mucin standard for use in both the Muc2 and Muc5ac ELISAs, and because of potential differences in assay kinetics of the subtype-specific antibodies. Therefore, the ratio of intracellular to secreted mucin for each subtype was examined, with this ratio greater for Muc2 than for Muc5ac. This suggests that less Muc2 than Muc5ac is secreted under constitutive circumstances, leaving Muc2 stores within the cells.

To determine if expression of the mucin subtypes can be modulated differentially in response to inflammation, GPTE cells were exposed to a mixture of pro-inflammatory cytokines (TNF-α, IL-1β, and IFN-γ or "cytomix") for 4 hrs. This exposure was carried out after a baseline mucus collection and a subsequent 8-hr rest period. Arbitrarily setting the level of constitutive mucin secretion (media only) for each subtype at 100%, secretion of Muc2 increased more than 100% over constitutive levels following cytokine stimulation, while no significant change in Muc5ac secretion was observed (Fig. 6b). In contrast, after setting the constitutive intracellular amount of Muc2 and Muc5ac (media only) at 100%, intracellular production of both mucin subtypes increased similarly following cytokine exposure, showing a 50% elevation over constitutive levels (Fig. 6c). These findings suggest inflammation may induce release of pre-formed Muc2 from GPTE cells, while at the same time stimulating the cells to increase intracellular mucin stores of both Muc2 and Muc5ac.

### Muc2 and Muc5ac gene expression changes with cytokine exposure

To determine if Muc2 and Muc5ac gene expression patterns correlate with mucin protein expression following exposure to pro-inflammatory cytokines, GPTE cells were exposed to cytomix over a time course of 0 to 12 hrs and total RNA was extracted from the cells. There appeared to be an overall trend toward an increase in steady-state levels of both Muc2 and Muc5ac mRNAs by 8 hrs of exposure (Fig. 6d); however, this increase was not statistically significant (p > 0.05 by ANOVA) due to variability between cultures. By contrast, at the earlier 4-hr time point, cytokine exposure induced a significant increase in Muc2 mRNA when compared to Muc5ac mRNA; Muc5ac mRNA remained at pre-exposure levels. Thus, while the increase in both mRNAs was ultimately similar, induction of Muc5ac gene expression seemed to lag behind that of Muc2. It may be that the early increase in Muc2 mRNA is a secondary consequence of the cytomix-induced preferential secretion of stored Muc2, rather than a direct induction of Muc2 transcription by the cytokines. These results suggest that exposure to pro-inflammatory cytokines can induce an increase in Muc2 gene expression in GPTE cells, with some increase in Muc5ac mRNA also possible with extended exposure. Such increases in gene expression could play a role in regulating the cumulative increase in intracellular Muc2 and Muc5ac observed in these cells following exposure to pro-inflammatory cytokines (Fig. 6c).

## Discussion

In this study, we have continued to develop molecular tools applicable to the study of mucus production in a guinea pig model, particularly targeting mucin protein expression. Specifically, we report the successful creation of a monoclonal antibody to guinea pig Muc2 (C4). In addition, we have optimized a commercially available anti-human MUC5AC monoclonal antibody (45M1) for detection of mucin, likely Muc5ac, from guinea pig. These antibodies have been employed to examine the expression of guinea pig Muc2 and Muc5ac during constitutive secretion from airway epithelial cells and following exposure of these cells to inflammatory cytokines. The discovery that the differential expression of two mucins can be readily measured opens avenues to molecular mechanistic studies which could not be done using previously reported anti-guinea pig mucin antibodies which most likely target the extensive, non-specific carbohydrate moieties on multiple mucin types [42].

Mucin specificity of each monoclonal antibody was examined using ELISA, Western blot, and IHC. Previous experimentation with 45M1 has led to its acceptance as MUC5AC-specific [36,43,44]; however, further proof was needed to establish the C4 monoclonal antibody as specific for Muc2. We therefore determined whether C4 and 45M1 exhibitted differential staining in mucous cell-containing tissues, and whether C4 could distinguish differentially between sections from murine small intestine and stomach. It has been established that secretions from these tissues are Muc2 and Muc5ac-dominant, respectively [15].

By immunohistochemistry, the C4 antibody recognized the content of specific cells within the murine intestinal epithelium (Fig. 2a), while the 45M1 antibody did not stain similarly in nearby sections from the same tissue (Fig. 3a). Additionally, no C4 staining was observed outside of the epithelial layer (Fig. 2a). Since the intestinal epithelium is rich in Muc2, and the C4 antibody was raised against a peptide expressed from a portion of the guinea pig Muc2 cDNA, the antibody is likely recognizing Muc2 in this tissue while the 45M1 antibody is not. The antibodies were also found to hybridize differentially to Western blots containing mucus secretions from guinea pig intestinal and stomach tissue (Figs. 2b and 3b). Secretions from guinea pig intestinal and stomach tissue were also separated by electrophoresis and then transferred to duplicate Western blots which were hybridized to either the C4 or the 45M1 antibodies. These antibodies hybridized differentially, with the C4 antibody hybridizing to very high molecular weight proteins in the intestinal secretions while the 45M1 antibody hybridized to large proteins in the stomach secretions that were a different size than the observed C4-reactive bands. Thus, as the intestine is known to be Muc2-rich and the stomach is Muc5ac-rich, we conclude that the C4 antibody and the 45M1 antibody are recognizing different mucins, likely Muc2 and Muc5ac, respectively. A similar pattern of specificity was also observed in ELISAs, with the anti-Muc2 antibody (C4) preferentially recognizing secretions from intestine, while the 45M1 antibody preferentially recognized stomach secretions. Both antibodies were found to label specific cells within the airway epithelium of guinea pig lung tissue, with little to no staining in underlying cells, endothelium or alveolar tissue (Fig. 4).

Taken together, these results indicate the two antibodies recognize different mucins, and that it is likely the C4 antibody recognizes Muc2 due, in part, to the manner in which the antibody was generated. Although we have not proven definitively that 45M1 recognizes Muc5ac, it does recognize a mucin abundant in airway epithelium and stomach tissue that is different in size from the mucin recognized by C4 (Figs. 3b and 4).

Use of the C4 and 45M1 antibodies has now provided the first complete picture of Muc2 and Muc5ac production and secretion in guinea pig airway epithelial cells. Kondo et al. have previously demonstrated intracellular Muc5ac protein production in differentiated guinea pig tracheal epithelial cells *in vitro *[45]. Additionally, we have previously examined expression of the mRNAs corresponding to these two mucins in guinea pig [25], determining that the Muc2 message predominates over the Muc5ac message in RNA isolated from GPTE cells. Data from our current study indicate that Muc2 and Muc5ac are present at the extracellular, intracellular, and mRNA levels in GPTE cells. Although it is difficult to ascertain relative amounts of Muc2 and Muc5ac glycoproteins due to differences in assay kinetics and lack of coordinated mucin standards, examination of the ratio of intracellular to extracellular mucin for each of the mucin subtypes is informative. When cells are growing without exposure to cytokines, Muc2 is detected as an abundant intracellular reserve with less Muc2 secreted constitutively; this is in contrast to Muc5ac which is detected in similar intracellular and extracellular amounts in such cells. Immunohistochemistry of lung tissue further supports the abundant presence of both Muc2 and Muc5ac within airway epithelial cells in the guinea pig.

There also appears to be a differential mucin response to pro-inflammatory mediators (TNFα, IL-1β, and IFNγ) in GPTE cells, with these cytokines stimulating an apparent increase in secreted Muc2, without a detectable increase in secreted Muc5ac. Interestingly, TNFα and IL1-β are known to each increase mucin secretion from airway epithelial cells independently [46,47], while INF-γ has been shown to have an inhibitory effect on mucus stimulation in mice [48]. Even though INF-γ and TNFα /IL1-β have counter-regulatory effects, these proteins are nevertheless all found in inflamed airways [49], and have been shown to collectively upregulate iNOS expression [50] which has been shown to upregulate mucin secretion [51]. Thus, while attempting to better mimic the complex inflammatory milieu found in a chronically-inflamed airway, we have found that this mixture of cytokines does modulate Muc2 secretion. Future studies will be needed to determine the contribution of the specific cytokines to the modulation of mucin expression in GPTE cells.

While we readily detected Muc2 in guinea pig tissue and GPTE cells, other studies examining mucin expression in bronchial or tracheal tissue, or epithelial cells from these tissues, in rat, mouse, horse or humans portray Muc5ac and/or Muc5b as the dominantly-expressed mucins both in healthy and in altered airways. In these studies, Muc2 is often detected minimally, or not at all [26,29,40,52-54]. One explanation for the lack of detection of Muc2 in airway secretions has been that it is insoluble [37,38,55]. Since neuraminidase had little affect on the ability of C4 to recognize Muc2 in our study (Fig. 5a), this might suggest the C4 antibody can detect O-glycosylated and non-glycosylated Muc2 similarly; however, this interpretation does not fully take into account the differential solubilities of these two mucin forms. Thus, the apparent increase in cytokine-induced Muc2 secretion might be due to a greater abundance of the more soluble, O-glycosylated Muc2 being secreted. A change in post-transcriptional processing of MUC2 has been noted in inflamed colonic mucosa from patients with Crohn's disease or ulcerative colitis. In that study, the inflammation-associated alteration in post-transcriptional modification of MUC2 yielded molecules with decreased sulphation whose presence was correlated to increased reaction with an anti-MUC2 antibody [56].

Thus, the finding that intracellular Muc2 is readily detectable in guinea pig tracheal epithelial cells, both constitutively and following exposure to pro-inflammatory cytokines, is novel. Furthermore, while Muc2 mRNA is already known to be enhanced by exposure to TNFα [25], our findings provide the first example of increased Muc2 secretion in GPTE cells in response to inflammatory stimuli. This high level constitutive and inducible Muc2 expression in guinea pig may vary from that observed in other mammalian species, including humans. It is additionally possible that different mucins respond to airway inflammation differently depending on the species. Such a possibility suggests the need to fully characterize which mucins respond maximally to inflammatory stimuli when a new animal model of mucus hypersecretion is being developed.

Regulatory mechanisms governing differential expression of specific mucin subtypes can exist at transcriptional and/or translational levels. It has been shown, for example, that inflammatory stimulation can augment the MUC5AC message while MUC5B levels remain unchanged in the human airway-derived cell lines A549 and NCI-H292 [52]. One mechanism by which MUC5AC expression can be upregulated involves modulation of mRNA stability. Such regulation has been demonstrated in A549 and normal human bronchial epithelial (NHBE) cell cultures, where the inflammation-associated protease, neutrophil elastase, induced an increase in MUC5AC message stability [57] via a signaling mechanism involving secondary production of reactive oxygen species (ROS) [13]. Transcriptional regulation of *MUC5AC *has also been demonstrated in epithelial cell lines, where ROS-dependent activation of an AP-1 response element upstream of the *MUC5AC *gene occurs after cigarette smoke exposure [58]. In addition, MUC2 transcripts have been observed in both mucin-expressing and non-expressing cells [59], suggesting regulation of MUC2 expression can also occur at the level of protein translation. In our study, we observe yet another potential level of differential mucin regulation by detecting an increase in apparent Muc2 secretion induced by cytokine stimulation, while the level of secreted Muc5ac remains unchanged. This apparent differential secretion occurs even though cytokine-induced effects on the intracellular protein production and the long-term mRNA levels of the two mucin subtypes do not appear to be regulated differentially. This finding supports the theory that mucin subtypes may be secreted differentially as a form of post-translational regulation. Alternatively, post-translational modification of mucins may be differentially regulated. Thus, the increased reaction of secreted Muc2 with the C4 antibody could be due to a cytokine-induced change in Muc2 glycosylation, while no such change in Muc5ac occurs.

Evidence from previous studies has demonstrated that mucin secretion occurs through coordinated cycles of phosphorylation/dephosphorylation of the PKC-substrate, MARCKS [60], in a mechanism which induces mucin granule movement toward the plasma membranes. Assuming this pathway is non-selective for a particular mucin subtype, the mucin granules themselves may be packaged differentially with regard to mucin subtypes. While differential secretion of specific mucin subtypes in the absence of differential transcriptional or translational control has not been reported previously, it has been suggested that different mucin granules within a single goblet cell may contain structurally distinct mucin subpopulations [61]. The differential regulation of mucin secretion from such mucin-specific granules in response to pro-inflammatory stimuli might be an important mechanism whereby the properties of airway mucus can be altered. Such property changes could provide greater protection to the underlying epithelial cells, but might also result in deleterious effects on lung function with prolonged airway inflammation.

In summary, we have used newly developed molecular tools to examine expression of Muc2 and Muc5ac in mucus collected from guinea pig tissues and GPTE cells. Specifically, a novel mouse monoclonal antibody was raised against guinea pig Muc2 apomucin, and it, along with a commercially-available antibody to MUC5AC, were optimized for specific recognition of mucin from guinea pig by ELISA, Western blot, and IHC. Using GPTE cells, it was found that pro-inflammatory stimulation with a cytokine mixture of TNF-α, IL-1β, and IFN-γ, stimulated increased secretion of Muc2, but not Muc5ac, while intracellular protein and long-term mRNA expression increased similarly for both mucin subtypes. These findings highlight differences in regulation of specific mucins within the same organism, as well as suggest comparative differences with regard to which mucins are highly expressed, normally and in disease, among a variety of mammalian species. This differential regulation associated with inflamed airways may well influence the mucosal makeup, and, ultimately, the physical properties of airway mucus in a manner that could affect clearance and gas exchange deleteriously.

## Abbreviations

cDNA = complementary deoxyribose nucleic acid; DMEM = Dulbecco's modified Eagle's medium; ELISA = enzyme linked immunosorbent assay; FBS = fetal bovine serum; GPTE = guinea pig tracheal epithelial; HRP = horseradish peroxidase; IFN-γ = interferon γ; IHC = immunohistochemistry; IL-1β = interleukin 1β; IPTG = isopropyl-1-thio-β-D-galactopyranoside; mRNA = messenger ribonucleic acid; OD = optical density; PBS = phosphate buffered saline; SDS-PAGE = sodium dodecyl sulfate polyacrylamide gel electrophoresis; RT-PCR = reverse transcriptase polymerase chain reaction; SEM = standard error of the mean; TNF-α = tumor necrosis factor α.

## Competing interests

KBA and LDM are members of the Scientific Advisory Board of BioMarck Pharmaceuticals, Ltd.

## Authors' contributions

BC conducted the majority of the research experiments, performed the statistical analysis, and drafted the manuscript. AC contributed to the neuraminidase protocol, antibody purification, and IHC studies as well as assisted with manuscript preparation. YL performed the foundation study and contributed to the design of this study. MM contributed to the design of the foundation and current study, and was instrumental in providing funding. KBA and LDM conceived of the antibody study, and participated in its design and coordination. In addition, LDM conceived and designed the secretion studies and was responsible for finalizing the manuscript. All authors have read and approved the final manuscript.
